# Integrating bioinformatics and experimental validation to reveal a novel VRK score as a prognostic and therapeutic biomarker in hepatocellular carcinoma

**DOI:** 10.3389/fimmu.2025.1614702

**Published:** 2025-09-17

**Authors:** Zhihao Fu, Dufei Liu, Menglin Chen, Guochao Zhong, Zhibo Zhao, Junhua Gong, Xin Dai, Jiejun Hu, Degong Jia, Lve Cheng, Dong Cai, Jianping Gong

**Affiliations:** ^1^ Department of Hepatobiliary Surgery, The Second Affiliated Hospital of Chongqing Medical University, Chongqing, China; ^2^ Department of Hepatobiliary and Pancreatic Surgery, People’s Hospital of Zhengzhou University, Zhengzhou, Henan, China; ^3^ Institute of Clinical Pathology, Key Laboratory of Transplant Engineering and Immunology, National Health Commission (NHC), West China Hospital, Sichuan University, Chengdu, Sichuan, China; ^4^ Department of Kidney Transplantation, The First Affiliated Hospital of Zhengzhou University, Zhengzhou, Henan, China

**Keywords:** VRK, immune, therapy, bioinformatics, HCC

## Abstract

**Background:**

Vaccinia-related kinase (VRK) family genes play a multifunctional role in tumor development. However, the role of VRK family genes in hepatocellular carcinoma (HCC) requires further research. Moreover, the clinical potential of the VRK-related model remains unclear. The aim of this study is to construct a VRK-related model to predict HCC prognosis and therapeutic efficacy.

**Methods:**

The data of HCC patients were extracted from The Cancer Genome Atlas (TCGA), International Cancer Genome Consortium (ICGC), and Gene Expression Omnibus (GEO) databases. The single-sample gene set enrichment analysis (ssGSEA) algorithm was used to calculate the VRK score of each sample. Tumor IMmune Estimation Resource 2.0 (TIMER 2.0) and Tumor Immune Dysfunction and Exclusion (TIDE) were used to evaluate immune cell infiltration and the immune checkpoint response. pRRophetic was used for predicting drug sensitivity. CCK-8, colony formation, wound healing, transwell and xenograft assays were used to experimentally validate the biofunction of VRK2 in HCC.

**Results:**

We found that all VRK family genes were highly expressed in HCC. Compared with patients with low VRK scores, patients with high VRK1 or VRK2 expression in the TCGA, ICGC, and GSE14520 cohorts had poorer outcomes. Moreover, patients with a high VRK score in the TCGA, ICGC, and GSE14520 cohorts also had poorer outcomes. Importantly, Cox analysis revealed that the VRK score was a potential independent risk factor for HCC. Notably, TIMER2.0 and TIDE suggested that patients with high VRK scores had higher immune checkpoint response rates. Similarly, drug sensitivity analyses suggested that patients with high VRK scores were more resistant to sorafenib, paclitaxel, cisplatin, and gemcitabine. Finally, experimental validation revealed that VRK2 knockdown inhibited HCC development *in vitro* and *in vivo*.

**Conclusion:**

The VRK score was found to be a reliable indicator for predicting HCC prognosis and therapeutic efficacy. VRK2 is a potential therapeutic target for HCC.

## 1 Introduction

Primary liver cancer is a malignant disease with the sixth highest incidence and the third highest mortality worldwide (1). Hepatocellular carcinoma (HCC) accounts for approximately 80% of primary liver cancer cases (2). Although HCC treatment methods have significantly advanced, the 5-year survival rate remains unsatisfactory (3–5), partly due to the lack of effective prognostic and therapeutic biomarkers. Recently, increasing evidence has shown that the construction of prognostic signatures and screening of biomarkers on the basis of bioinformatics can effectively predict the outcomes of HCC patients and the efficacy of drug therapy (6–8). The signatures of the disulfidptosis-related lncRNAs were successfully used to assess the immune status and chemotherapy drug sensitivity of HCC patients, which provided novel insights into precision therapy (9). These studies provide a theoretical basis for further exploration of HCC data and screening of reliable biomarkers through bioinformatics methods. Larger sample sizes and lower costs are advantages to using this methodology. However, current prognostic models and biomarkers have limitations. The most important limitation is that these models are constructed by algorithms that produce a specific risk coefficient value and divide HCC patients into high- and low-risk groups to evaluate the outcomes of HCC patients and effects of treatment strategies. Ignoring the different cohorts produces different coefficient values due to the heterogeneity of the samples. Therefore, there is a subjective tendency to choose a coefficient value from the training set as the unified standard. Therefore, further development of novel models and biomarkers for evaluating the outcomes of HCC patients and therapeutic effects of treatment strategies is essential.

Vaccinia-related kinase (VRK) family genes include VRK1, VRK2, and VRK3. This family of genes exhibits serine/threonine protein kinase activity (10). The crucial roles and functions of serine/threonine protein kinases, including MAPK, AKT, and mTORC, in solid tumors have been widely investigated and reported (11, 12). In clinical samples of HCC, phosphorylation of mTORC and AKT has been observed to be associated with poor prognosis (13, 14). Functionally, phosphorylation of mTORC and AKT promotes proliferation and cell cycle regulation in HCC cells (15, 16). Given the established importance of serine/threonine protein kinases, VRK family genes with serine/threonine protein kinase activity likely contribute significantly to the development of HCC. Previous studies have shown that VRK family genes play important roles in HCC progression. *In vitro* VRK1 knockdown suspended the cell cycle and inhibited proliferation in the setting of HCC (17). Similarly, VRK2 is enriched in sorafenib-resistant HCC cells, and VRK2 knockdown overcomes sorafenib resistance in HCC (18). This evidence supports the importance of VRK family genes in HCC. Unfortunately, studies focusing on the role of VRK family genes in the outcomes of HCC patients and efficacy of treatment methods are limited. Therefore, systematic exploration of novel prognostic signatures based on VRK family genes is urgently needed to evaluate HCC prognosis and treatment efficacy.

In this study, we systematic investigated the expression and prognosis of VRK family genes in HCC and different subgroups. Next, we established a novel prognostic signature (the VRK score) independent of subjective selection bias using the single-sample Gene Set Enrichment Analysis (ssGSEA) algorithm. Importantly, we verified the robustness of our prognostic signature in three independent HCC cohorts. Furthermore, our prognostic signature successfully predicted the efficacy of drug treatment in HCC patients. Moreover, we experimentally verified that VRK2 knockdown inhibited HCC proliferation *in vitro* and *in vivo*. Overall, our study highlights the importance of VRK family genes and identifies a novel signature and biomarkers for assessing HCC prognosis and drug efficacy.

## 2 Materials and methods

### 2.1 Data collection and processing

For the Cancer Genome Atlas (TCGA) data (TCGA-LIHC), mRNA expression transcriptome profiles (transcripts per kilobase of exon model per million mapped reads format, TPM) and corresponding clinical information were obtained using the ‘TCGAbiolinks’ package (version 2.28.4) (19). For International Cancer Genome Consortium (ICGC) data (ICGC-LIRI-JP), mRNA expression transcriptome profiles and corresponding clinical information were acquired from the ICGC database (https://dcc.icgc.org/). For Gene Expression Omnibus (GEO) data (GSE14520), mRNA expression transcriptome profiles and corresponding clinical information were acquired from the GEO database (https://www.ncbi.nlm.nih.gov/geo/). All HCC samples without complete clinical information (such as TNM stage, sex, age, survival status, and time) were removed. Finally, the TCGA cohort (T = 365, N = 50), ICGC cohort (T = 203, N = 177) and GEO cohort (T = 239, N = 239) were created.

### 2.2 VRK score construction and independent prognostic analysis

The single-sample Gene Set Enrichment Analysis (ssGSEA) algorithm was used with the ‘GSVA’ package (version 2.0.6) to calculate the VRK score of each HCC sample. The optimum cutoff values were selected with the ‘survival’ package (version 3.8.3). Univariate and multivariate Cox analyses were performed with SPSS software (version 27). Nomograms and calibration curves were produced with the ‘rms’ package (version 7.0).

### 2.3 Immune and tumor microenvironment analysis

Immune cell infiltration analysis based on the 6 algorithms (XCELL, TIMER, MCPCOUNTER, QUANTISEQ, EPIC, and CIBERSORT) was performed using the TIMER2.0 database (http://timer.comp-genomics.org/) (20). The ssGSEA algorithm was used with the ‘GSVA’ package (version 2.0.6) to calculate the scores of immune cells and the function of each HCC sample. Next, immune checkpoint blockade response prediction and corresponding Tumor Immune Dysfunction and Exclusion (TIDE) analyses were performed using the TIDE database (http://tide.dfci.harvard.edu/).

### 2.4 Gene mutation analysis and drug sensitivity prediction

Gene mutation analysis and tumor mutation burden (TMB) analysis of the TCGA cohort were performed with the ‘maftools’ package (version 2.22.0). Drug sensitivity prediction was subsequently performed with the ‘pRRophetic’ package (version 0.5).

### 2.5 Cell culture and transfection

MHCC97H cells were obtained from the National Collection of Authenticated Cell Cultures. The cells were cultured in DEME (HyClone, China) containing 10% fetal bovine serum (Gibco, USA) and 1% penicillin–streptomycin solution (Solarbio, China) at 37°C with 5% CO_2_. Small interfering RNAs (siRNAs) were designed and constructed by GenePharma, and short hairpin RNA (shRNA) lentivirus was constructed from siRNA#1 by ViGene Biosciences. For siRNA transfection, cells (2x10^5^/well) were seeded into a 6-well plate and transfected with Lipofectamine™ 3000 (L3000015, Thermo Fisher Scientific, USA) according to the manufacturer’s instructions. For shRNA transfection, cells (1x10^5^/well) were seeded into a 6-well plate and transfected according to the manufacturer’s instructions. After 48 hours, the efficacy of VRK2 knockdown was verified by the quantitative real-time reverse transcription PCR (qRT–PCR). The sequences of the siRNAs can be found in **Supplementary Table S1**.

### 2.6 Cell proliferation and half-maximal inhibitory concentration assays

For the CCK-8 assays, cells (2000/well) were seeded into a 96-well plate. Next, a CCK-8 kit (ZP328-1, Beijing Zoman Biotechnology, China) was used to detect the absorbance of the samples at 0, 24, 48, 72, and 96 hours. Briefly, 10 μl of CCK-8 solution was added to each well, followed by incubation at 37°C for 2 hours. Then, the absorbance values were detected at 450 nm. For the half-maximal inhibitory concentration assays, cells (5000/well) were seeded into a 96-well plate and treated with indicated concentrations of cisplatin for 24 hours. Then, 100 μl fresh complete medium containing 10 μl CCK-8 solution was added to each well, followed by incubation at 37°C for 2 hours. Subsequently, the absorbance values were detected at 450 nm. For the colony formation assays, the cells (500/well) were seeded into a 6-well plate and cultured for 14 days. Next, 4% paraformaldehyde solution was used to fix the cells for 20 minutes at room temperature, followed by staining for 20 minutes at room temperature with 0.1% crystal violet solution. The number of cells was determined with ImageJ software (version 1.53). Cisplatin (M2223) was purchased from AbMole.

### 2.7 Cell metastasis assays

For the wound healing assays, the cells were seeded into a 6-well plate. When cell fusion reached 95%, a wound was generated by scraping the middle of the plate with a 200 μl sterile pipette tip, after which the medium was replaced with serum-free medium. After 48 and 72 hours, the migration distance was measured with ImageJ software (version 1.53). For transwell assays, 24-well transwell chambers with 8 μm pores and Matrigel (354480, Corning, USA) were used. Briefly, cells (1x10^4^/well) were seeded into the top compartment with 250 μl of serum-free medium, and 500 μl of complete medium was added to the bottom compartment. After 30 hours, the cells that had passed through the filter were fixed with 4% paraformaldehyde for 30 minutes at room temperature, followed by staining for 20 minutes at room temperature with 0.1% crystal violet solution. The number of cells was determined with ImageJ software (version 1.53).

### 2.8 Gene set enrichment analysis

GSEA was performed by the GSEA soft (version 4.3.2). The hallmark gene sets were obtained from the molecular signatures database (https://www.gsea-msigdb.org/gsea/msigdb/index.jsp). The number of permutations is 500. The pathways with FDR <0.25 and P <0.05 were considered to have differences between high and low VRK2 groups.

### 2.9 Animal model

Five-week-old male BALB/c nude mice were purchased from GemPharmatech and housed in a specific pathogen-free environment with a 12-h light/dark cycle and controlled temperature and humidity, and food and water were provided ad libitum. MHCC97H cells (2x10^6^/mouse) were subcutaneously injected into the mice. The tumor volume was measured every 5 days. The tumor volume formula was as follows: volume = 0.5 × longest diameter × (shortest diameter)^2^. After 15 days, the mice were euthanized, and the tumors were fixed with 4% paraformaldehyde for 2 days, then embedded in paraffin. All operations on laboratory animals were performed in accordance with the NIH Guide for the Care and Use of Laboratory Animals and were approved by the Animal Care and Use Committee of West China Hospital, Sichuan University (20240815015).

### 2.10 Immunohistochemistry

Paraffin-embedded tumor tissue sections were deparaffinized, repaired with 0.01 M citric acid buffer at 95°C for 20 minutes and incubated overnight at 4°C with a Ki-67 antibody (1:10000, 27309-1-AP, Proteintech, China). Then, the sections were incubated with secondary antibody (PV-6000, ZSGB-BIO, China) for 1 hour at room temperature after being washed with TBS three times. The IHC results were quantified with ImageJ software (version 1.53).

### 2.11 Western blotting

Protein lysates were prepared by RIPA buffer (P0013B, Beyotime, China) and run on sodium dodecyl sulfate-polyacrylamide gels for electrophoresis (ZD304A-2, Zoman, China). Separated proteins were then transferred to the polyvinylidene fluoride (PVDF) membranes (ISEQ00005, Millipore, USA). The membranes were blocked with 5% skim milk and incubated with the primary antibody in the blocking buffer (overnight at 4°C) followed by horseradish-peroxidase-conjugated secondary antibodies (Proteintech) for 1 h at room temperature. The blots were developed by performing the enhanced chemiluminescence detection reagents on the membranes and the signals were detected by the ECL blotting analysis system (4AW011-100, 4abio, China). Beta-actin in Western blotting was used as the endogenous loading control. Anti-VRK2 antibody (1:1000; Proteintech; 12946-1-AP), anti-AKT1 antibody (1:1000; HUABIO; ET1609-47), anti-pAKT1 antibody (1:1000; HUABIO; ET1607-73), anti-RPS6 antibody (1:1000; Proteintech; 80208-1-RR), anti-pRPS6 antibody (1:1000; Proteintech; 29223-1-AP), anti-beta-actin antibody (1:1000; HUABIO; M1210-2), goat anti-rabbit IgG (1:10000; ZSGB-BIO; ZB-2301) and goat anti-mouse IgG (1:10000; ZSGB-BIO; ZB-2305)were used.

### 2.12 Statistical analysis

All data are presented as the means ± standard deviations (SDs). The comparison of quantitative data between the two groups was performed using Student’s t test (normal distribution) or the Mann–Whitney test (nonnormal distribution). The comparison of quantitative data among three or more groups was performed using one-way or two-way ANOVA with Bonferroni correction. Count data were compared using the chi-square test. Correlation analysis was performed using Spearman correlation analysis. The prognostic analysis based on Kaplan–Meier survival curves was completed using the log-rank test. All statistical analyses were completed with R (version 4.4.2), SPSS (version 27), and GraphPad Prism (version 10.2.3) software. The ‘ggplot2’ package (version 3.5.1) and GraphPad Prism (version 10.2.3) were used to create plots. *P* < 0.05 was considered statistically significant.

## 3 Results

### 3.1 Expression levels of VRK family genes

**Figure 1:**
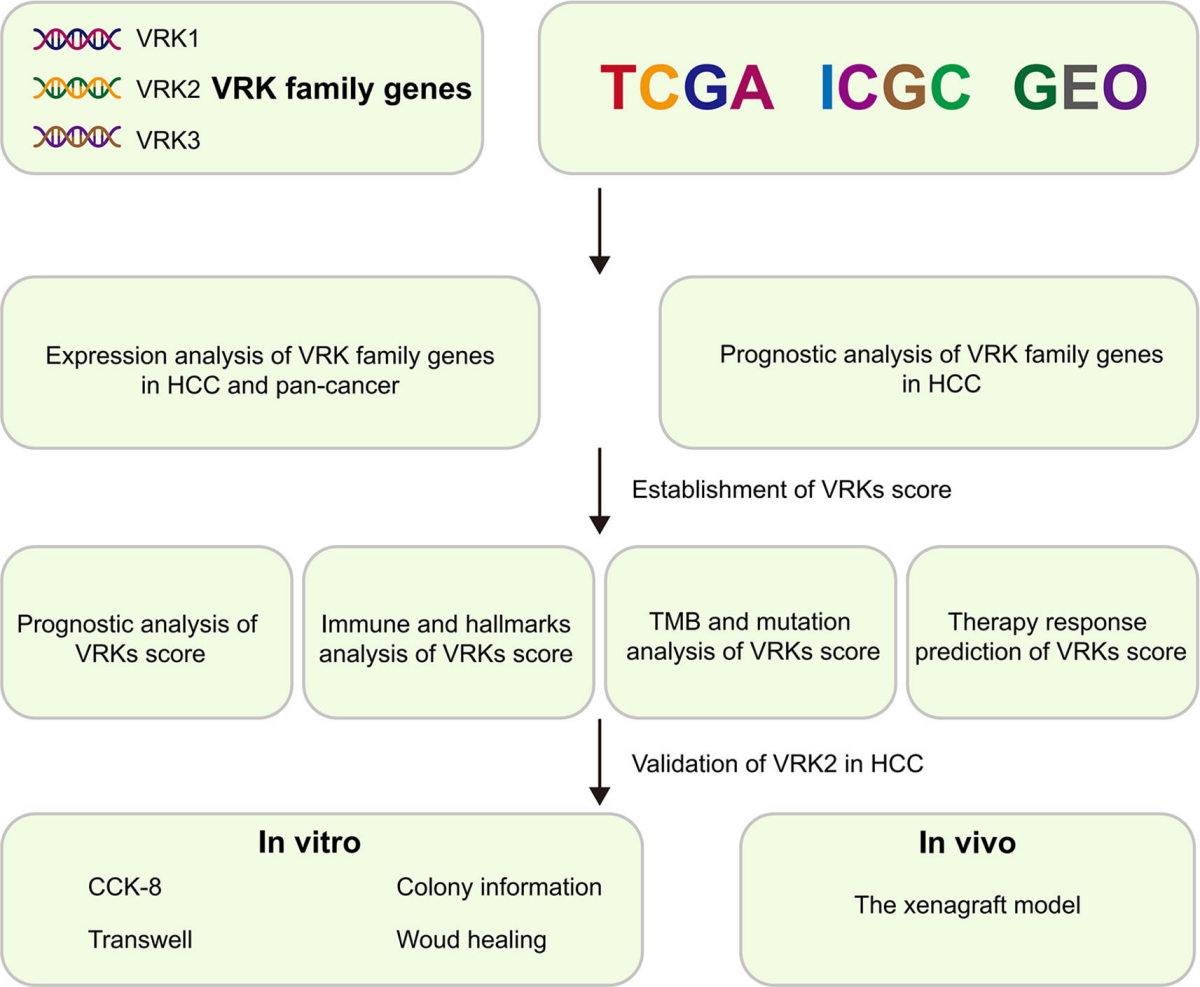
Flowchart of this study.

A comprehensive flow diagram is shown in **Figure 1**. To comprehensively understand the expression distribution of VRK family genes in cancer, we first used the TIMER2.0 database to explore the expression of VRK family genes in 22 cancers. As expected, the expression of VRK family genes was consistent across cancers. In breast, bile duct, esophageal, lung, and colon cancers, VRK family genes were highly expressed in tumors, whereas the expression of VRK family genes was lower in kidney and thyroid cancers (**Supplementary Figure S1**).

**Figure 2:**
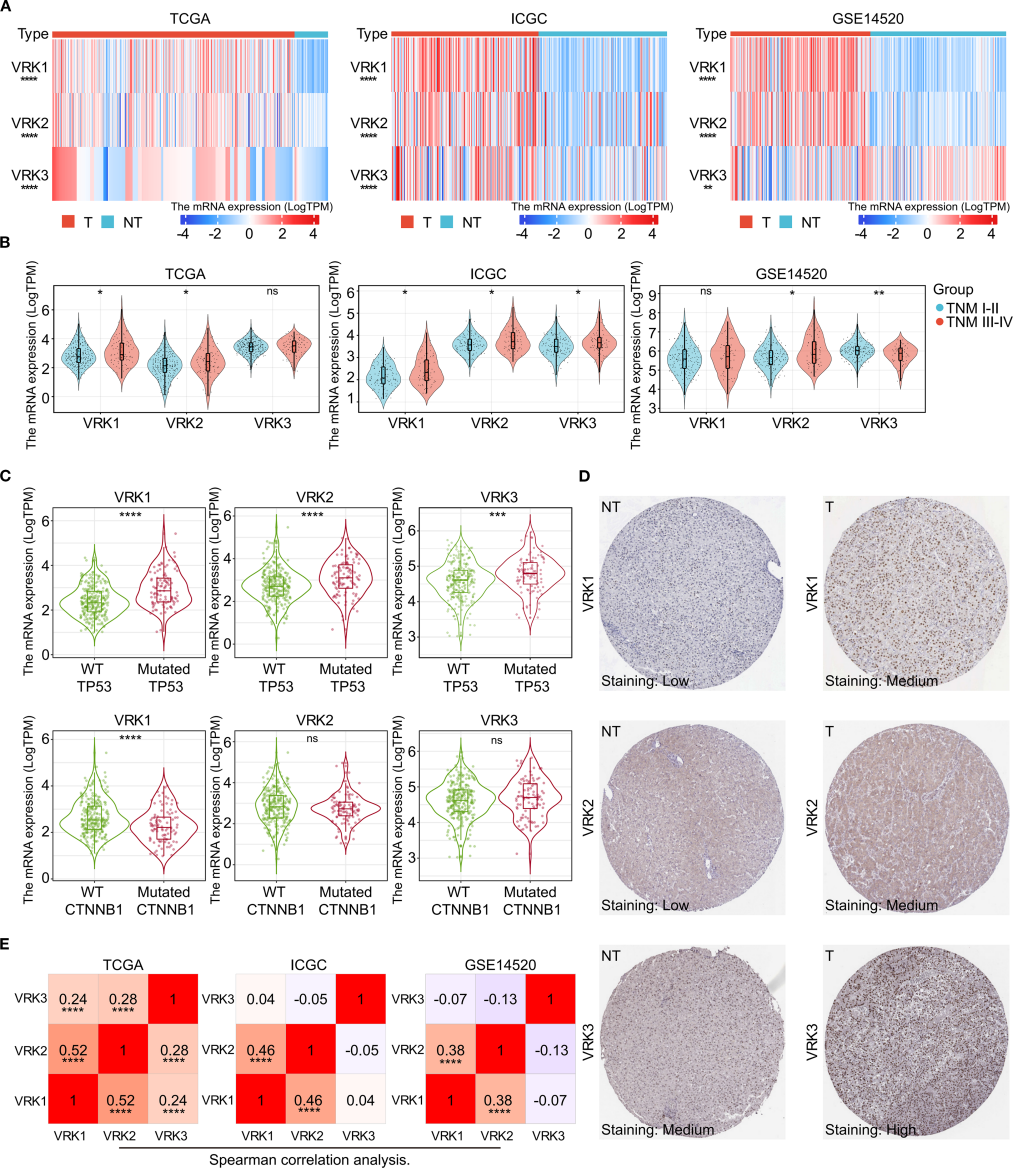
Differential mRNA and protein expression of VRK family genes. **(A)** mRNA expression heatmap of VRK family genes between tumor and nontumor tissues in the TCGA, ICGC, and GSE14520 cohorts. **(B)** mRNA expression of VRK family genes between the TNM stages I-II and III-IV groups in the TCGA, ICGC, and GSE14520 cohorts. **(C)** mRNA expression of VRK family genes in the wild-type and mutated groups in the TCGA, ICGC, and GSE14520 cohorts. **(D)** Representative IHC images of VRK family genes in tumor and nontumor tissues from the HPA database. **(E)** Correlation analysis of VRK family genes in the TCGA, ICGC, and GSE14520 cohorts. T, tumor; NT, nontumor; WT, wild type. *P<0.05, **P<0.01, ***P<0.001, ****P<0.0001.

**Figure 3:**
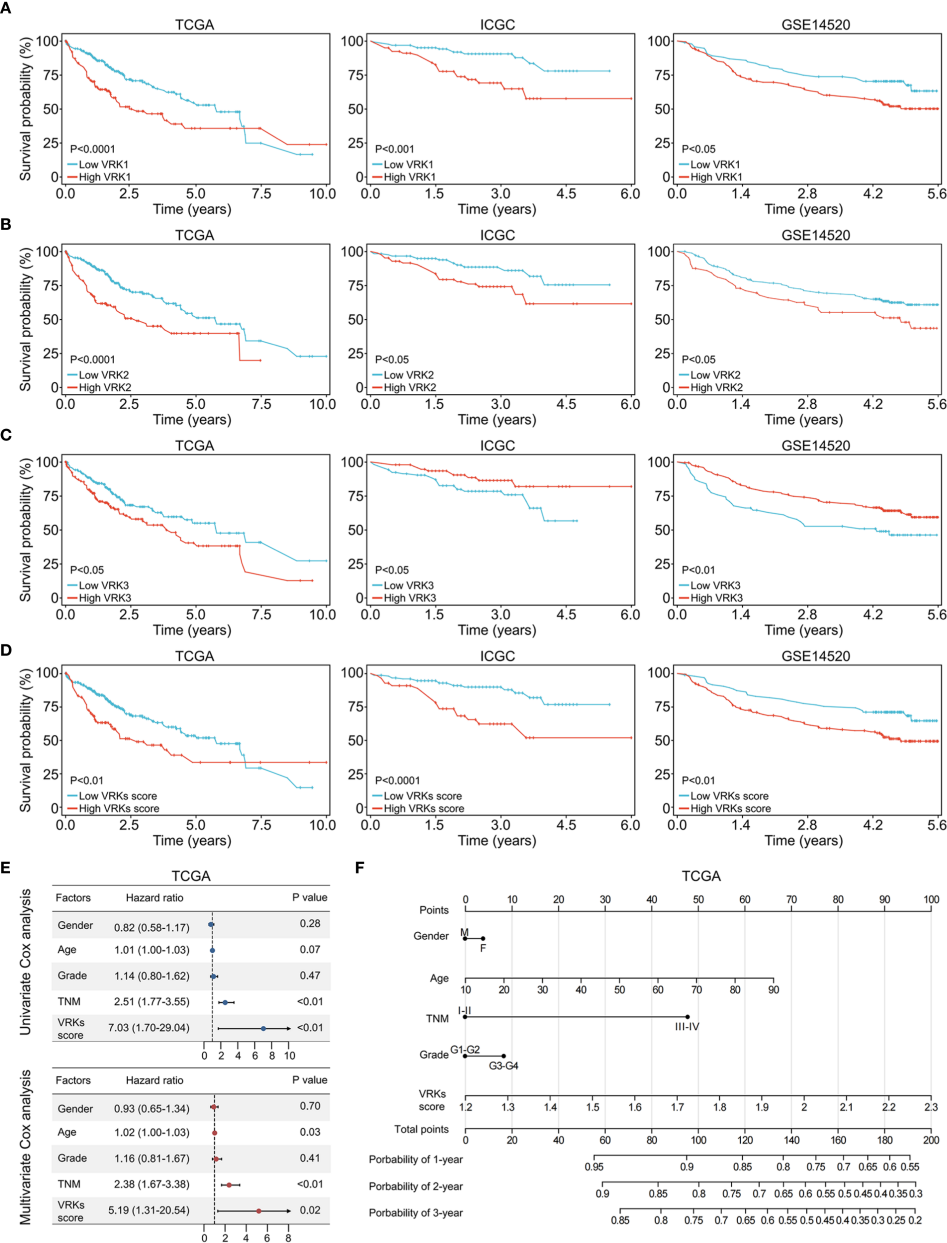
Prognostic analysis of VRK family genes and VRK scores. **(A)** The overall survival of HCC patients with high and low VRK1 expression in the TCGA, ICGC, and GSE14520 cohorts. **(B)** The overall survival of HCC patients with high and low VRK2 expression in the TCGA, ICGC, and GSE14520 cohorts. **(C)** The overall survival of HCC patients with high and low VRK3 expression in the TCGA, ICGC, and GSE14520 cohorts. **(D)** The overall survival of HCC patients with high and low VRK scores in the TCGA, ICGC, and GSE14520 cohorts. **(E)** Univariate and multivariate Cox analysis of the VRK score and other clinical features in the TCGA cohort. **(F)** The nomogram of the VRK score and other clinical features in the TCGA cohort.

Next, we comprehensively explored the expression of VRK family genes in HCC. All three independent HCC cohorts (TCGA, ICGC, and GSE14520) exhibited high VRK gene family expression (**Figure 2A**). The subgroup analysis further revealed that only VRK2 expression was closely associated with TNM stage in the three HCC cohorts, whereas VRK1 expression was associated with TNM stage in the TCGA and ICGC cohorts and VRK3 expression was associated with TNM stage in the ICGC and GSE14520 cohorts (**Figure 2B**). Furthermore, we did not observe an obvious association of VRK family genes with sex or age (**Supplementary Figures S2A, B**). TP53 and CTNNB1 are commonly mutated genes in HCC and are closely associated with HCC progression (21). Therefore, we also compared the expression of VRK family genes between the wild-type and mutated groups in HCC patients in the TCGA cohort. Notably, the expression of VRK family genes was greater in the mutated TP53 groups, whereas the expressions of VRK2 and VRK3 were not significantly different between the CTNNB1 wild-type and mutated groups (**Figure 2C**). Therefore, the expression of VRK family genes appeared to be more closely associated with TP53 than with CTNNB1. Furthermore, we examined the protein expression of VRK family genes in HCC using the HPA database. Consistently, the IHC results revealed that the protein expression of VRK family genes was increased in tumor tissues (**Figure 2D**). Additionally, we evaluated correlations between VRK family genes using Spearman correlation analysis. In the three HCC cohorts, VRK1 expression was significantly and positively correlated with VRK2 expression, whereas VRK3 expression was not obviously correlated with VRK1 or VRK2 expression (**Figure 3E**). Further, we also analyzed the correlations between VRK family genes and immune cell infiltration in tumor microenvironment (TME) using Spearman correlation analysis (**Supplementary Figures S2C–E**). Overall, VRK family genes were highly expressed in most cancers, and the expression of VRK family genes in HCC was closely associated with TNM stage and TP53 mutation.

### 3.2 Establishment and independent prognostic analysis of the VRK score

Considering the high expression of VKR family genes in HCC, we next explored the effect of VRK family genes on the outcomes of HCC patients. First, we used the ‘survival’ package to automatically obtain the optimal cutoff values for VRK1, VRK2, and VRK3. Next, HCC patients were divided into high- and low-expression groups based on the cutoff values of the three genes. HCC patients with high VRK1 or VRK2 expression had poorer overall survival than patients with low VRK1 or VRK2 expression in all three independent HCC cohorts (**Figures 3A, B**). Interestingly, patients with high VRK3 expression had better overall survival than patients with low VRK3 expression in the ICGC and GSE1520 cohorts, although the results in the TCGA cohort were the opposite (**Figure 3C**).

Therefore, we aimed to construct a novel prognostic signature to evaluate HCC prognosis based on the VRK family gene expressions. Given the inconsistent prognostic results of VRK3 expression in the three cohorts, we used VRK1 and VRK2 for construction of the signature. With the ssGSEA algorithm, we calculated the VRK score of each HCC patient in the three cohorts and divided the patients into high- or low-VRK score groups using the ‘survival’ package. As expected, patients with high VRK scores had poorer overall survival than patients with low VRK scores did in all cohorts (**Figure 3D**). Furthermore, univariate and multivariate Cox analyses suggested that the VRK score was not inferior to the TNM stage as an independent prognostic risk factor for HCC in the TCGA cohort (**Figure 3E**). Moreover, we constructed a nomogram to assess HCC prognosis visually based on the VRK score (**Figure 3F**). To further verify the robustness of the VRK score, we also examined whether the VRK score was not inferior to the TNM stage as an independent prognostic risk factor for HCC in the other two cohorts. As expected, we found that the VRK score remained an independent prognostic risk factor in the ICGC and GSE14520 cohorts. Additionally, we plotted nomograms of the two cohorts to assess HCC prognosis based on the VRK score (**Supplementary Figures S3A–D**). Moreover, calibration curves were used to evaluate the accuracy of the nomograms (**Supplementary Figure S3E**). Overall, these results support the potential of the VRK score as an independent prognostic risk factor in HCC patients.

### 3.3 TME and immune checkpoint blockade response analysis of the VRK score

**Figure 4:**
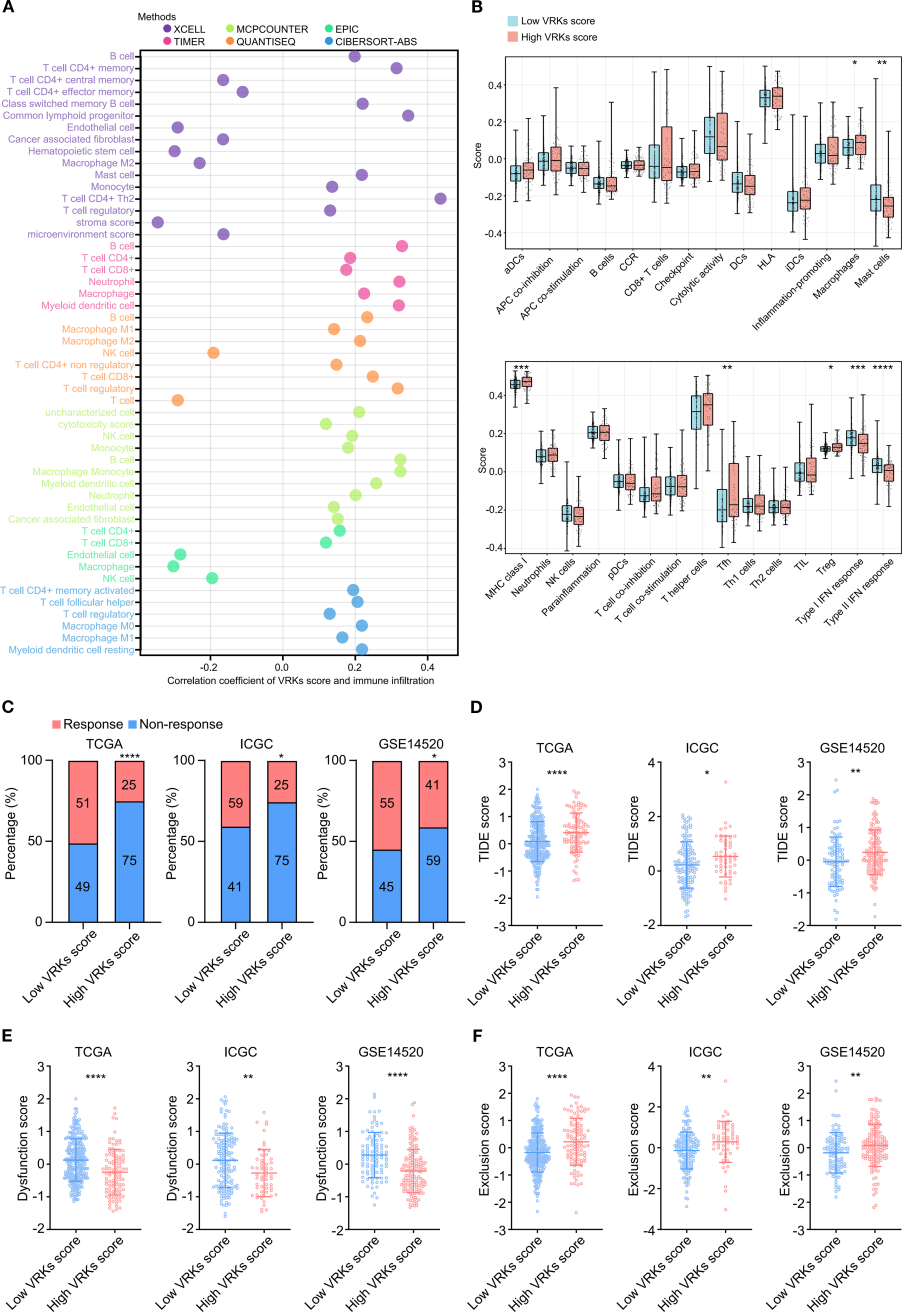
Immune microenvironment and immune checkpoint response analysis of the VRK score. **(A)** Heatmap of immune cell infiltration and correlation with the VRK score in the TCGA cohort based on 6 algorithms. **(B)** The score based on single-sample Gene Set Enrichment Analysis (ssGSEA) of immune cell infiltration and function between the high- and low-VRK score groups in the TCGA cohort. **(C)** Immune checkpoint response rates of the high- and low-VRK score groups in the TCGA, ICGC, and GSE14520 cohorts. **(D)** TIDE scores of the high- and low-VRK score groups in the TCGA, ICGC, and GSE14520 cohorts. **(E)** Dysfunction scores of the high- and low-VRK score groups in the TCGA, ICGC, and GSE14520 cohorts. **(F)** Exclusion scores of the high- and low-VRK score groups in the TCGA, ICGC, and GSE14520 cohorts. NK, natural killer; DC, dendritic cell; CCR, chemokine receptor; HLA, human leukocyte antigen; MHC, major histocompatibility complex; Tfh, follicular helper T cell; Th, helper T cell; Treg, regulatory T cell; TIDE, tumor immune dysfunction and exclusion. *P<0.05, **P<0.01, ***P<0.001, ****P<0.0001.

Increasing evidence has shown that immune activity in the tumor microenvironment (TME) strongly affects HCC prognosis and treatment (22, 23). Therefore, we explored immune cell infiltration in the TME in the high- and low-VRK score groups. The heatmap depicting immune infiltration revealed substantial correlations between the VRK score and various immune cells, including B cells, T cells, macrophages, and NK cells (**Figure 4A**). Furthermore, we explored the differences in immune infiltration and function between the high- and low-VRK score groups using the ssGSEA algorithm. The results revealed that the high-VRK-score group was characterized by elevated levels of macrophages, follicular helper T cells (Tfh), and regulatory T cells (Treg), which were juxtaposed with decreased levels of mast cells and a decreased interferon (IFN) response (**Figure 4B**). In addition, a heatmap depicting immune infiltration in the ICGC and GSE14520 cohorts was used to verify the correlations between the VRK score and immune cells. Decreased levels of the IFN response were also observed in the ICGC and GSE14520 cohorts (**Supplementary Figures S4A–D**).

Considering the disparities in immune infiltration and function associated with high and low VRK scores, we then used TIDE analysis to predict the response to immune checkpoint blockade (such as anti-PD-1 therapy) in the VRK score subgroups. The ratios of response in the low-VRK-score group were greater than the ratios of response in the high-VRK-score group in the three HCC cohorts (**Figure 4C**). Patients with high VRK scores had higher TIDE and exclusion of T cell scores and lower T cell dysfunction scores than patients with low VRK scores (**Figures 4D–F**), suggesting that patients in the low-VRK-score group may be more likely to benefit from immune checkpoint blockade. Moreover, GSEA analysis revealed that the high-VRK-score groups were characterized by upregulation of E2F targets, G2M checkpoint, DNA repair, mitotic spindle, mTORC1 signaling, and MYC targets in three HCC cohorts (**Supplementary Figures S5A–C**). In conclusion, these results revealed that the VRK score may be a potential predictor of the response to immune checkpoint blockade in HCC patients.

### 3.4 Mutation, TMB, and drug susceptibility analysis of VRK scores

**Figure 5:**
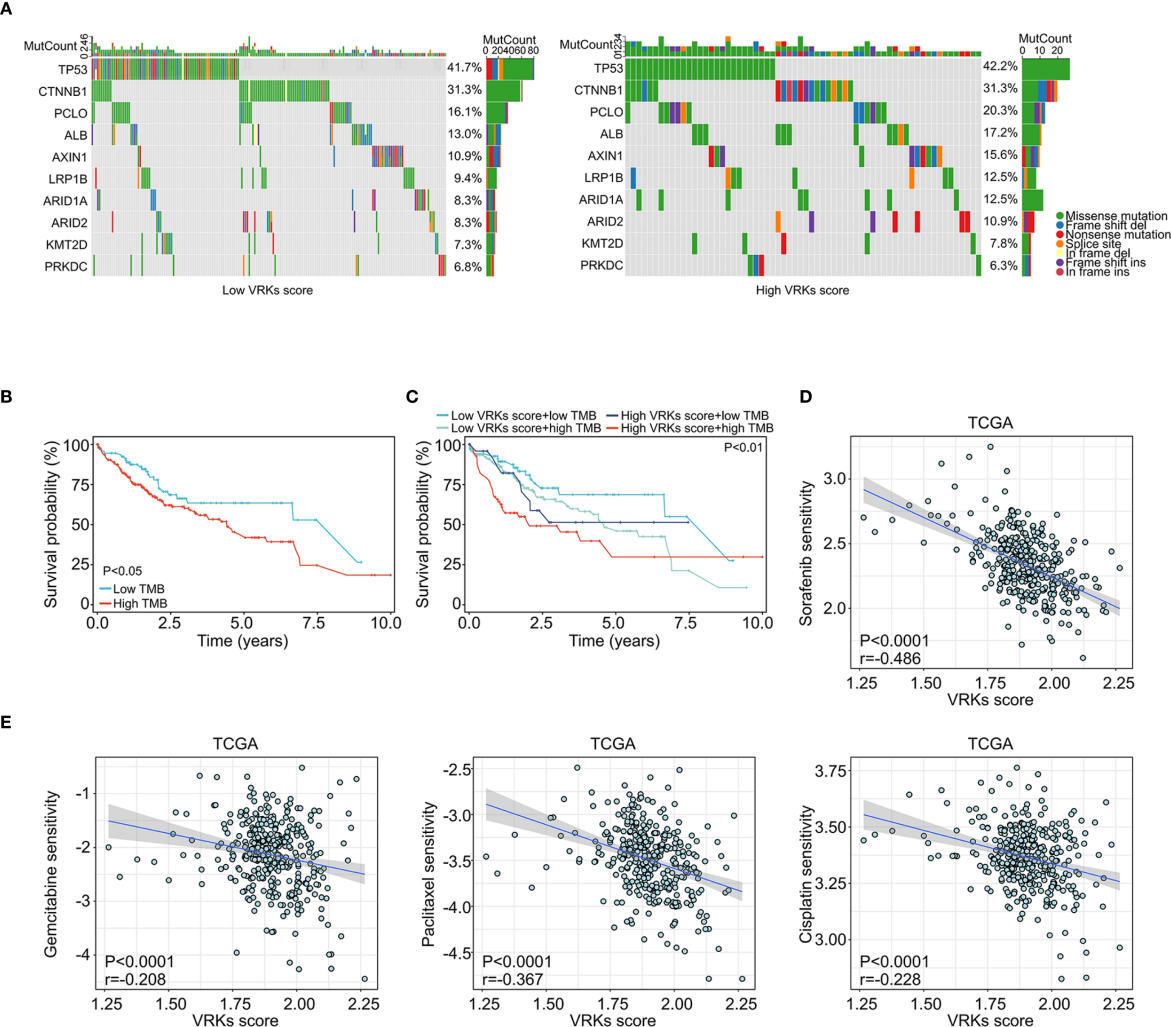
Mutation and drug sensitivity of the VRK score. **(A)** Waterfall plots showing the mutation frequency of common protumoral genes in HCC in the high- and low-VRK score groups in the TCGA cohort. **(B)** The overall survival of HCC patients with high and low TMB scores in the TCGA cohort. **(C)** The overall survival of HCC patients with VRK score combined with TMB score in the TCGA cohort. **(D)** The correlation analysis of the VRK score and sorafenib sensitivity in the TCGA cohort. **(E)** The correlation analysis of the VRK score and sensitivity to gemcitabine, paclitaxel, and cisplatin in the TCGA cohort. TMB, tumor mutation burden.

In addition to the tumor immune microenvironment, genetic mutations in tumors also affect HCC prognosis and treatment (24). Waterfall diagrams depicting gene mutation frequency revealed that TP53 and CTNNB1 are frequently mutated in both the high- and low-VRK score groups (**Figure 5A**). Increasing evidence has shown that the tumor mutational burden (TMB) reflects the gene mutation levels and prognosis of tumors (25). Accordingly, we calculated the TMB of each HCC patient in the TCGA cohort and found that patients with high TMB had poorer overall survival (**Figure 5B**). Furthermore, a high TMB combined with a high VRK score was associated with the poorest outcomes in HCC patients (**Figure 5C**).

Next, we assessed the sensitivity of other systemic therapy drugs beyond immune checkpoint inhibitors among the VRK score subgroups. Interestingly, patients with a low VRK score were more likely to be sensitive to sorafenib, gemcitabine, paclitaxel, and cisplatin (**Figures 5D, E**). These results were verified in the ICGC and GSE14520 cohorts (**Supplementary Figures S6A–C**). In summary, these findings demonstrated that the VRK score serves as a robust predictive biomarker for systemic therapy response in HCC patients.

### 3.5 VRK2 knockdown inhibited cell proliferation and metastasis in HCC

**Figure 6:**
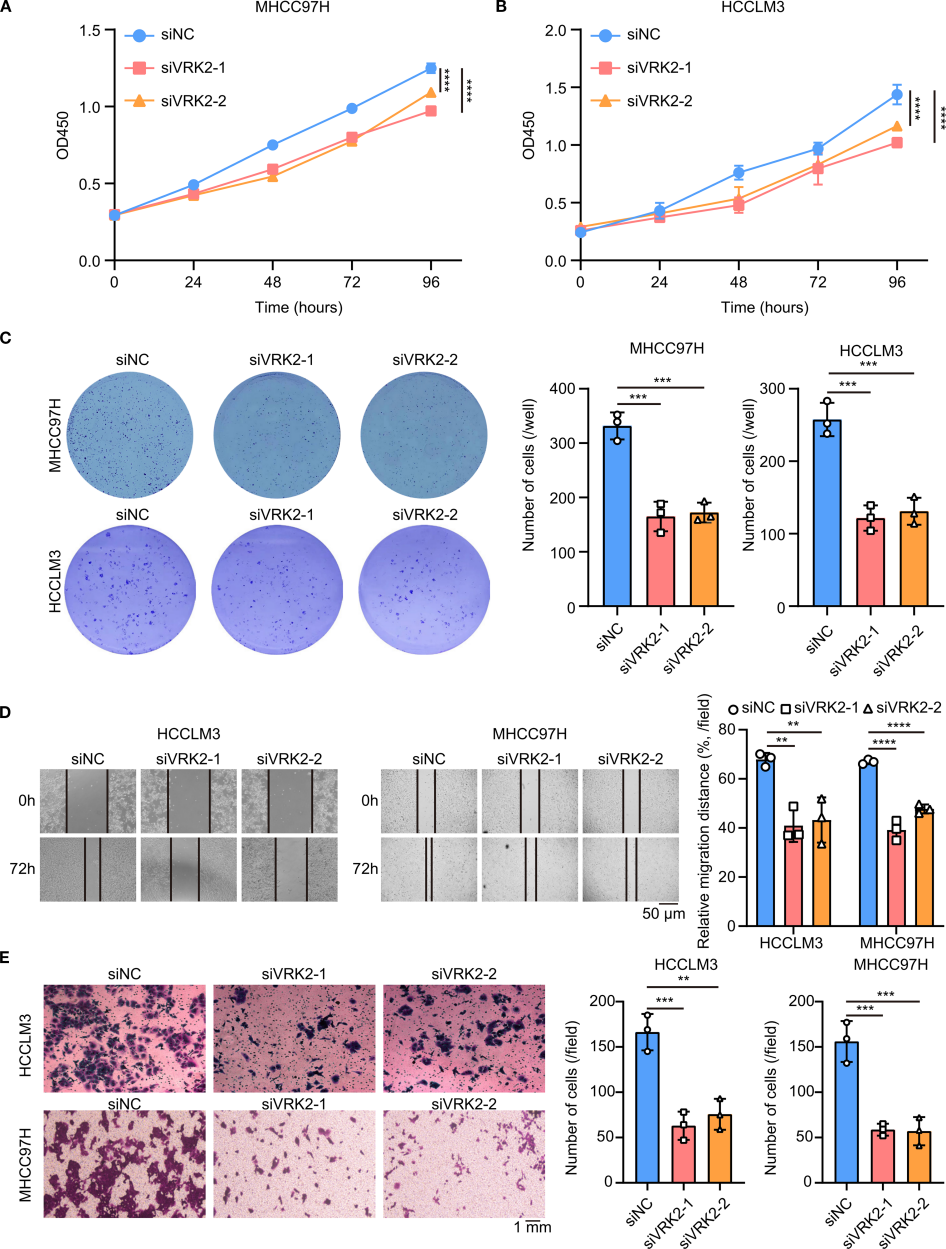
VRK2 knockdown inhibited cell proliferation *in vitro*. **(A, B)** CCK-8 results showing that VRK2 knockdown suppressed MHCC97H **(A)** and HCCLM3 **(B)** cell proliferation. **(C)** Colony formation results showing that VRK2 knockdown decreased the number of colonies formed by MHCC97H and HCCLM3 cells. **(D)** Wound healing results showing that VRK2 knockdown suppressed cell migration. **(E)** Transwell results showing that VRK2 knockdown inhibited cell invasion. siRNA, small interfering RNA. **P<0.01, ***P<0.001, ****P<0.0001.

As VRK2 was highly expressed in tumor tissues in all three HCC cohorts in this study, we further investigated the functional role of VRK2 in HCC. The western blot results revealed that the siRNA successfully knocked down VRK2 protein expression (**Supplementary Figure S7A**). The results of the CCK-8 and colony formation assays suggested that VRK2 knockdown inhibited the proliferation ability of MHCC97H and HCCLM3 cells (**Figures 6A–C**). Similarly, the results of the wound healing and transwell assays also indicated that VRK2 knockdown suppressed the migration and invasion ability of MHCC97H and HCCLM3 cells (**Figures 6D, E**). Then, we validated that VRK2 knockdown increased the sensitivity of HCC cells to cisplatin (CDDP) (**Supplementary Figure S7B**). Further, we performed the GSEA analysis and found that the PI3K/AKT/mTOR pathway was enriched in high VRK2 expression groups in three HCC cohorts (**Supplementary Figures S7C, D**). Importantly, western blot results verified that VRK2 knockdown decreased the phosphorylation levels of AKT1 and RPS6 (**Supplementary Figure S7E**). Additionally, we also found that patients with TNM staging III-IV had the poorer prognosis (**Supplementary Figures S7F, G**). Therefore, these results showed that VRK2 knockdown suppressed HCC proliferation and metastasis *in vitro*, as well as decreasing in phosphorylation levels of AKT1 and RPS6.

**Figure 7:**
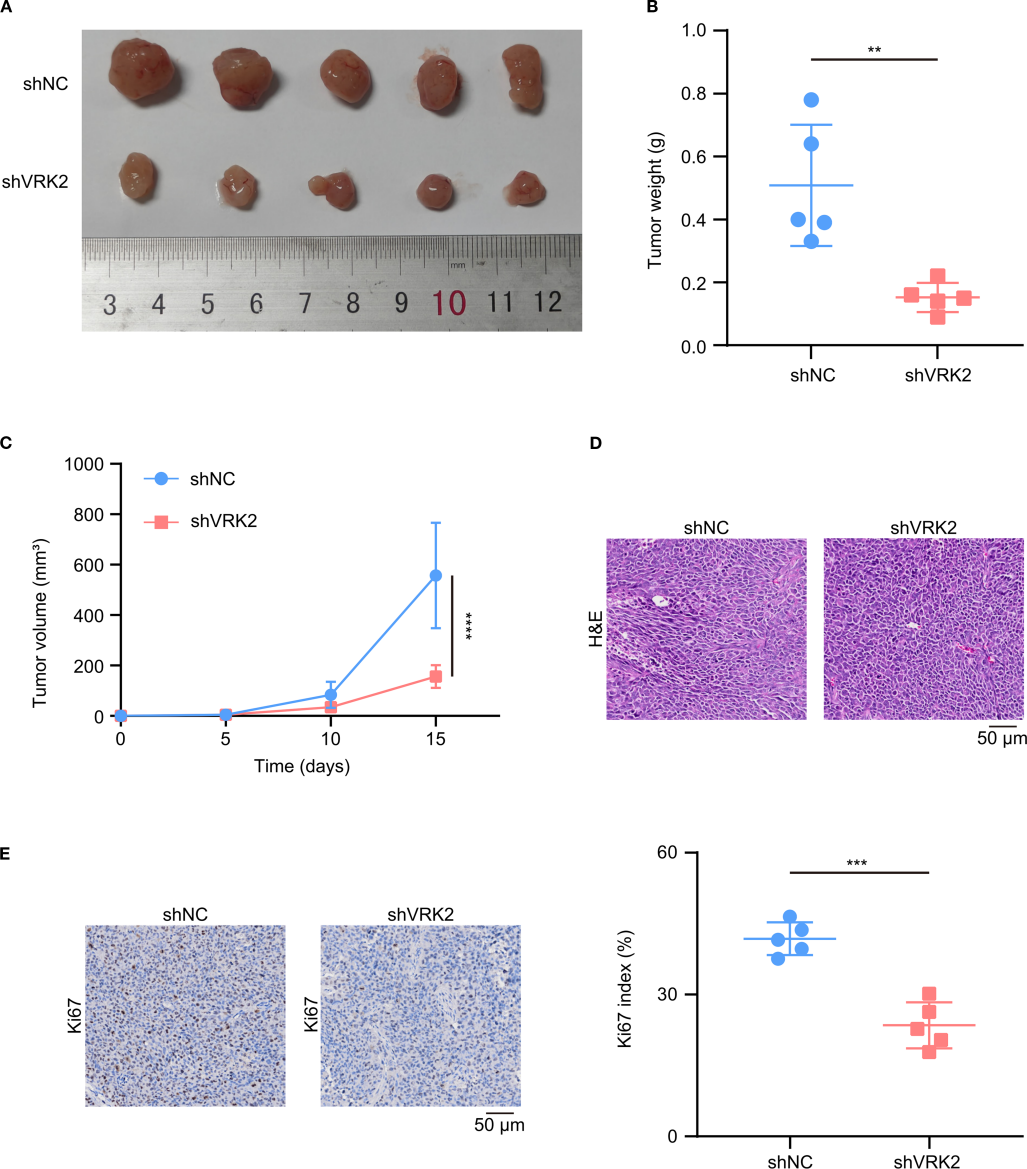
VRK2 knockdown inhibited cell proliferation *in vivo*. **(A)** Xenograft images of MHCC97H cells. **(B)** VRK2 knockdown decreased the tumor weight. **(C)** VRK2 knockdown decreased the tumor volume. **(D)** Representative H&E staining images of xenografts. **(E)** Representative Ki67 staining images (left panel) and statistical results (right panel) of the xenografts. H&E, hematoxylin and eosin. **P<0.01, ***P<0.001.

Next, we examined the effect of VRK2 knockdown on HCC *in vivo* (**Supplementary Figure S7H**). The xenograft tumor model verified that VRK2 knockdown inhibited tumor growth *in vivo*, accompanied by reductions in both tumor weight and volume (**Figures 7A–C**). H&E staining verified that the xenografts were tumors (**Figure 7D**). Additionally, Ki-67 staining revealed that VRK2 knockdown decreased tumor proliferation (**Figure 7E**). Taken together, these findings indicated that VRK2 knockdown inhibited HCC progression *in vitro* and *in vivo*.

## 4 Conclusion

Hepatocellular carcinoma (HCC), the predominant type of primary liver cancer, is a serious threat to an individual’s health and life due to its increasing morbidity and mortality (26). Recently, several models and signatures have been constructed for the prediction of the efficacy of treatment strategies and outcomes of HCC patients. Nevertheless, due to methodological limitations in modeling and tumor heterogeneity, the translational application of these models in clinical practice remains a significant challenge (7, 27, 28). Therefore, the identification of innovative biomarkers and signatures for predicting prognosis and therapeutic response holds paramount clinical significance for precision therapy in HCC. There are three key points to highlight in our study. First, we established a novel model independent of specific coefficient values from training sets using the single-sample Gene Set Enrichment Analysis (ssGSEA) algorithm. Second, the VRK score precisely predicts the outcomes and therapeutic responses of HCC patients. Finally, we experimentally confirmed that VRK2 knockdown inhibited HCC growth *in vitro* and *in vivo*.

Several prognostic models and molecular subtypes have been developed to predict the prognosis and treatment efficacy of solid tumors. For example, in breast cancer, stemness-related lncRNA signatures and disulfidptosis-related risk scores based on the least absolute shrinkage and selection operator (LASSO) algorithm are used to predict patient outcomes, stemness, and immunotherapy response (29, 30). Similarly, in lung cancer, radioresistance-related signatures also predict patient outcomes and immune status (31), identifying TOP2A, CDH3, ASPM, CENPF, SLC2A1, and PRC1 as potential detection biomarkers for early lung cancer (32). Additionally, in hepatocellular carcinoma (HCC), a three-gene prognostic model based on micro vessel invasion-related genes has been used to assess overall survival and recurrence-free survival (7). Although these prognostic models are established based on various proteins or noncoding RNAs, their modeling methods all rely on specific coefficient values generated by regression analysis of training sets. Due to the high heterogeneity of tumors, the clinical application of such methodological models may face challenges. Recently, modeling methods based on the ssGSEA algorithm for predicting patient outcomes and treatment efficacy have attracted widespread attention (33). This approach is independent of specific coefficient values and involves calculating the risk score based on the total RNA level of each sample. In fact, models based on the ssGSEA algorithm for evaluating tumor prognosis and treatment efficacy have been validated across cancers. For example, a high FOXO score indicates excellent immune activity in the microenvironment and sensitivity to drug treatment (34). Notably, our VRK score based on the ssGSEA algorithm not only matches previous prognostic models for predicting HCC prognosis and treatment efficacy but also demonstrates superior robustness while overcoming the limitations of previous models. Specifically, our research results indicated that the univariate and multivariate Cox regression analysis of the VRKs score in multiple cohorts all performed better than TNM staging. However, signatures from other researchers’ studies were not superior to TNM staging (35, 36). Meanwhile, our VRKs score was constructed through the ssGSEA algorithm while other studies’ signatures were constructed by Cox and LASSO algorithms (7, 35). Importantly, our VRKs score accurately predicted the response of patients to immune checkpoint blockade treatment, which is overlooked in the studies of others (35–37).

VRK family genes encode VRK1, VRK2, and VRK3 proteins with serine/threonine protein kinase activity (10). Previous studies have focused mainly on the roles and functions of VRK1 in solid tumors, whereas relatively few studies have focused on VRK2 and VRK3. In addition, an increasing number of studies have indicated that VRK family genes play crucial roles in the cell cycle, the DNA damage response, and metastasis in tumors (38, 39). These studies highlight the importance of VRK family genes in tumor development. Unfortunately, there is a lack of relevant studies on tumor prognostic models and molecular subtypes based on VRK family genes. Our study established a novel prognostic model based on VRK family genes and highlighted the importance of VRK family genes in cancer from a bioinformatics perspective. Notably, previous studies have shown that VRK2 can promote HCC metastasis and sorafenib resistance (19, 39). Our study further confirmed that VRK2 knockdown inhibited cell proliferation *in vitro* and *in vivo*. Moreover, we also found that the VRK score was positively associated with sorafenib resistance, which is consistent with the findings of Chen. Interestingly, the opposite results for VRK3 expression and prognosis were obtained in the ICGC and GSE14520 cohorts. To ensure the consistency of the VRK score and VRK family genes in the setting of HCC, we believe that VRK3 may not be suitable for inclusion as a modeling molecule. In fact, few studies have reported the roles and functions of VRK3 in solid tumors, including HCC.

To date, an increasing number of prognostic models and molecular subtypes have been developed for evaluating HCC prognosis and treatment efficacy (9, 23). Nevertheless, no model based on VRK family genes has been developed for HCC. In our study, a novel model was developed based on VRK family genes to predict HCC prognosis and treatment efficacy. With the increasing application of immune checkpoint blockade (ICB) therapy in combination with other drug therapies for HCC, it is critical to identify reliable biomarkers or assessment systems to predict therapeutic responses in HCC patients. Our study constructed a VRK score and successfully predicted ICB efficacy, as well as the efficacy of several drugs (sorafenib, paclitaxel, cisplatin, and gemcitabine) used for treating HCC. Importantly, these results can be verified across multiple independent HCC cohorts, confirming the robustness of the VRK score and providing theoretical support for further clinical translation. Considering that patients with high VRK scores have a poorer outcomes and efficacy of ICB and several drugs (sorafenib, paclitaxel, cisplatin, and gemcitabine) than patients with low VRK scores, HCC patients with high VRK family gene expressions and high VRK scores should be monitored closely.

However, there are several limitations in our study. First, our study was based on information from previous HCC cohorts, and no prospective study has been conducted to further validate our findings. Second, our study did not further investigate the molecular mechanisms of VRK family genes in the setting of HCC. In our study, a novel model was constructed to evaluate the outcomes of HCC patients and the therapeutic efficacy of HCC treatments, which we hope will provide a basis for the precise treatment of HCC.

## Data Availability

The original contributions presented in the study are included in the article/**Supplementary Material**. Further inquiries can be directed to the corresponding author/s.
