# Supplementary material for: Integrating bioinformatics and experimental validation to reveal a novel VRK score as a prognostic and therapeutic biomarker in hepatocellular carcinoma
Source: Front Immunol. 2025 Sep 17;16:1614702. doi: 10.3389/fimmu.2025.1614702 (PMC12484037; doi:10.3389/fimmu.2025.1614702)
Supplement: Supplementary file 1 [file Table1.docx]

Supplementary Material

# Supplementary Figures


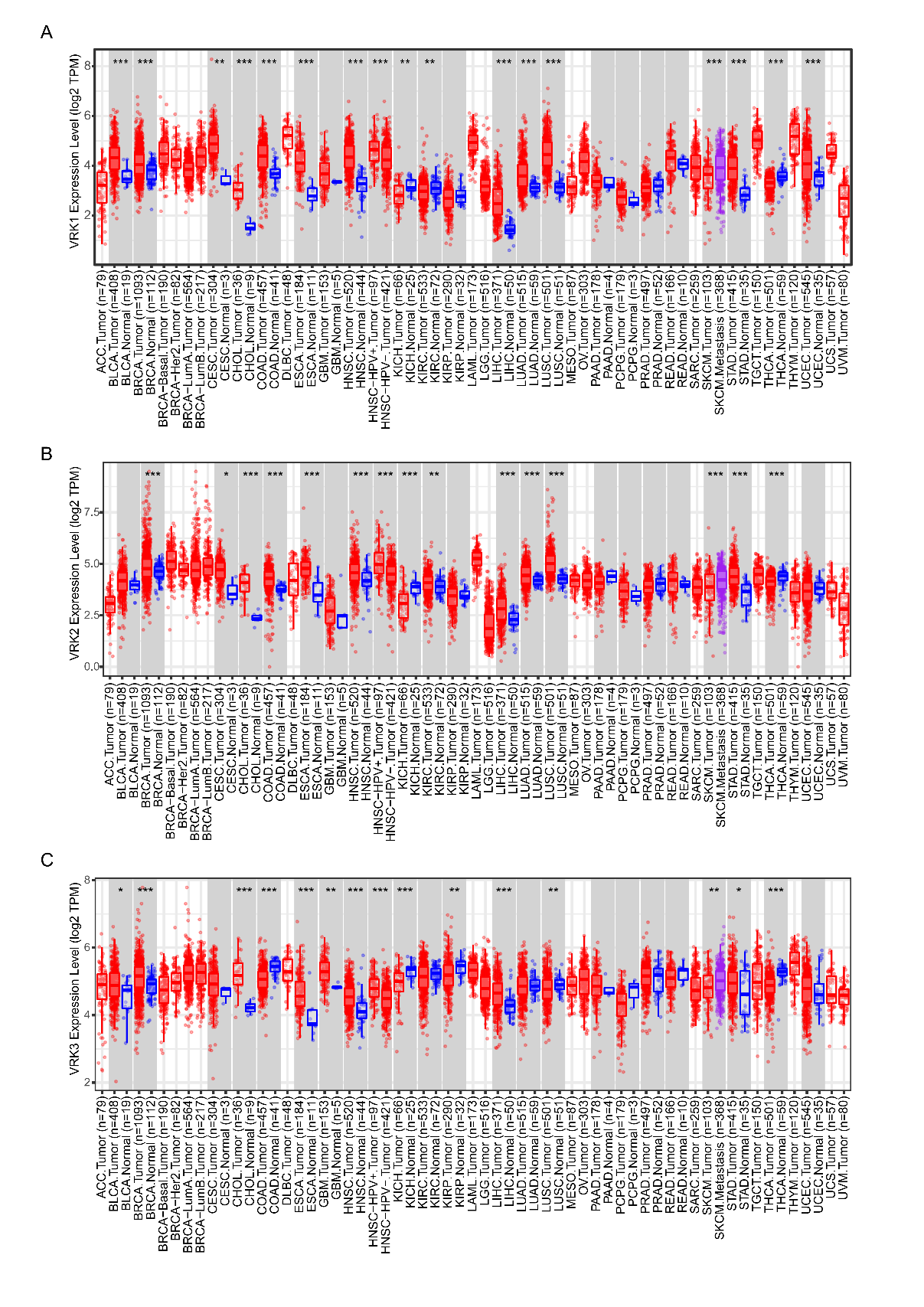


**Supplementary Figure 1.** The mRNA expression of VRK family genes in pan-cancer from the TIMER2.0 database. (A) The mRNA expression of VRK1 in pan-cancer. (B) The mRNA expression of VRK2 in pan-cancer. (C) The mRNA expression of VRK3 in pan-cancer. **P*<0.05, ***P*<0.01, ****P*<0.001, *****P*<0.0001.


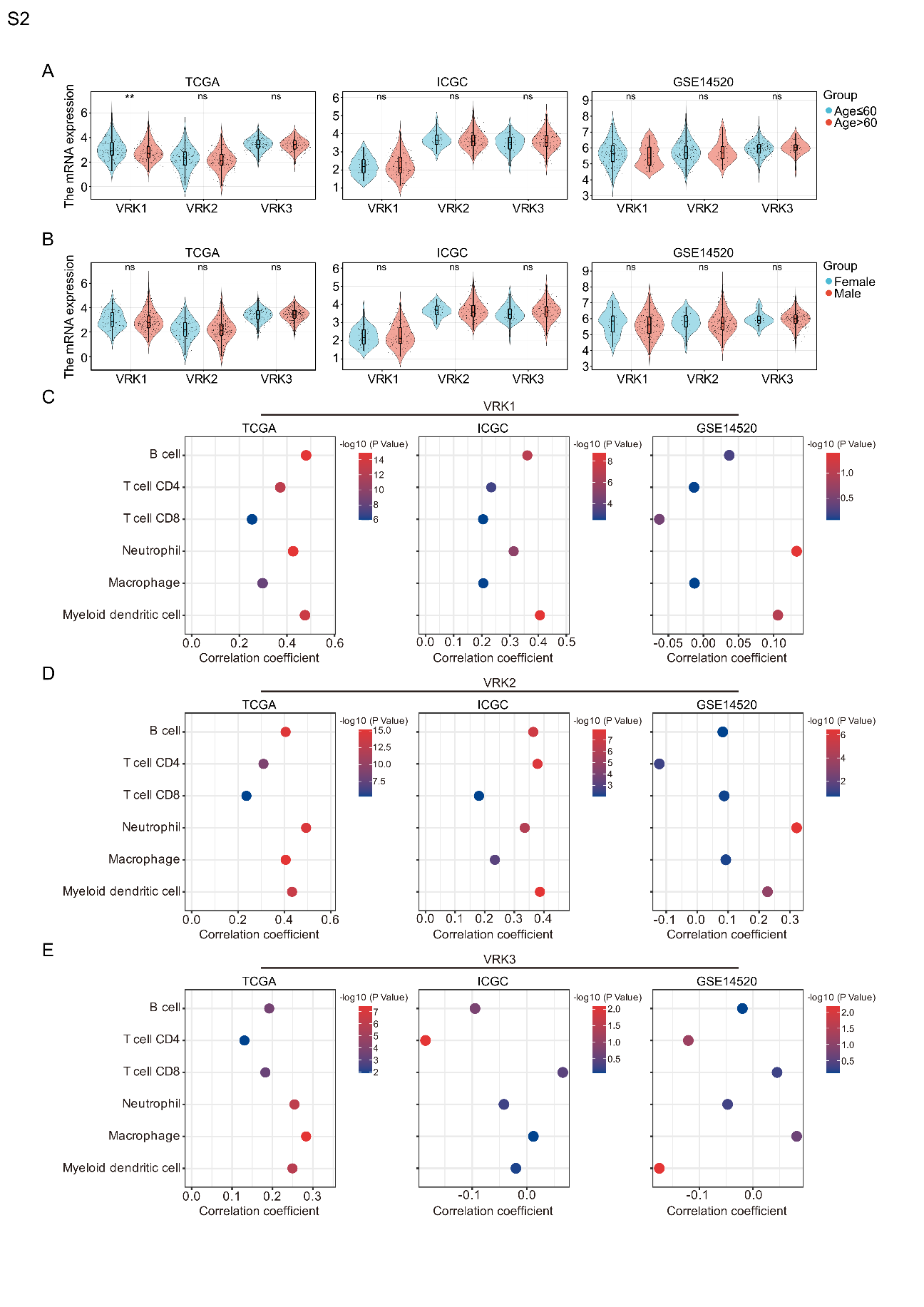


**Supplementary Figure 2.** The mRNA expression analysis of VRK family genes in different subgroups. (A) The mRNA expression analysis of VRK family genes in age subgroups. (B) The mRNA expression of VRK2 in pan-cancer. (C) The spearman correlation analysis between VRK1 and immune cells based on TIMER. (D) The spearman correlation analysis between VRK2 and immune cells based on TIMER. (E) The spearman correlation analysis between VRK3 and immune cells based on TIMER. ns, no significance, **P*<0.05, ***P*<0.01, ****P*<0.001, *****P*<0.0001.


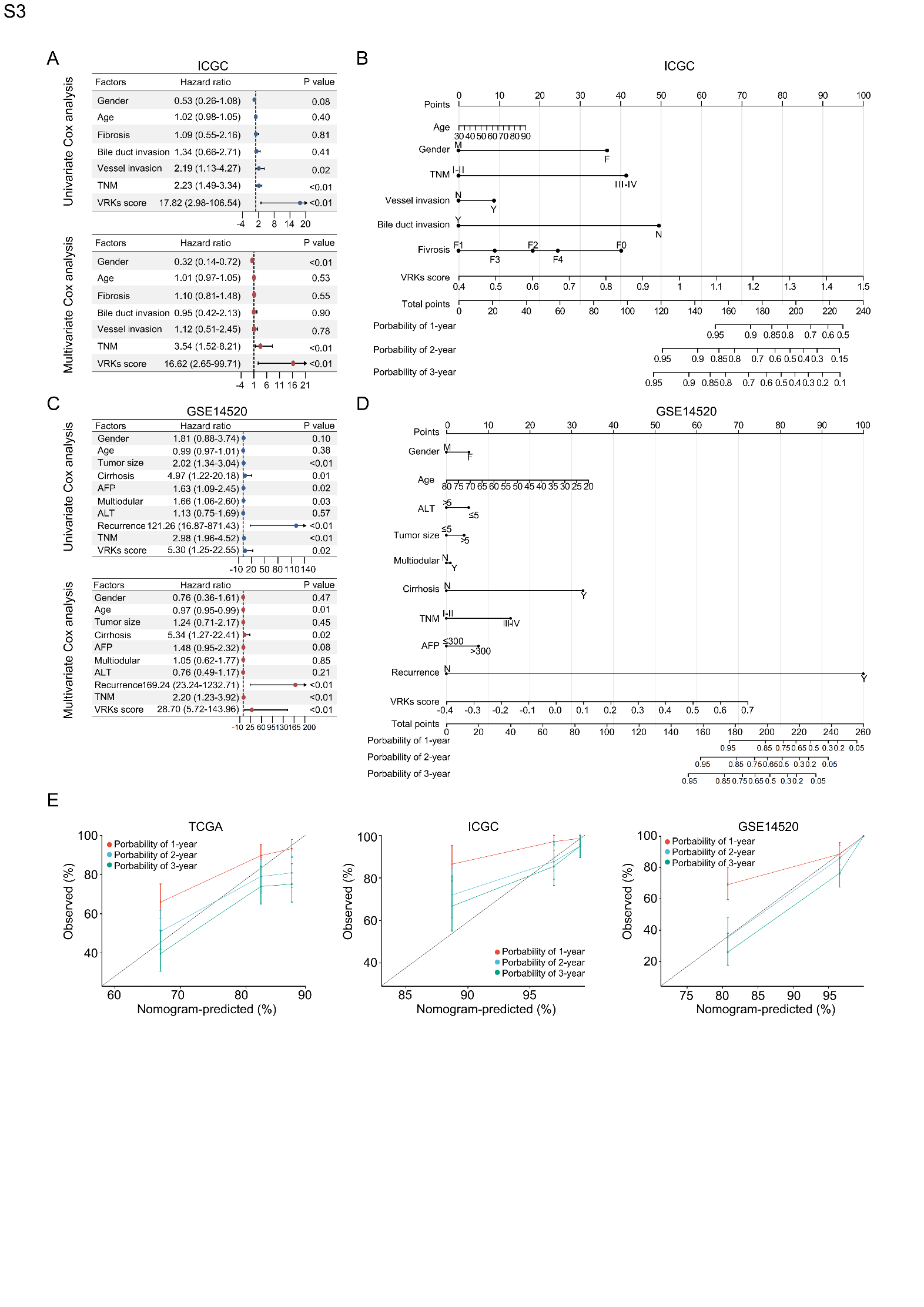


**Supplementary Figure 3.** The prognostic analysis of the VRK family genes and VRKs score. (A) Univariate and multivariate Cox analysis of the VRKs score and other clinical features in the ICGC cohort. (B) The nomogram of the VRKs score and other clinical features in the ICGC cohort. (C) Univariate and multivariate Cox analysis of the VRKs score and other clinical features in the GSE14520 cohort. (F) The nomogram of the VRKs score and other clinical features in the GSE14520 cohort. (E) Calibration curves of VRKs score in TCGA, ICGC and GSE14520 cohorts.


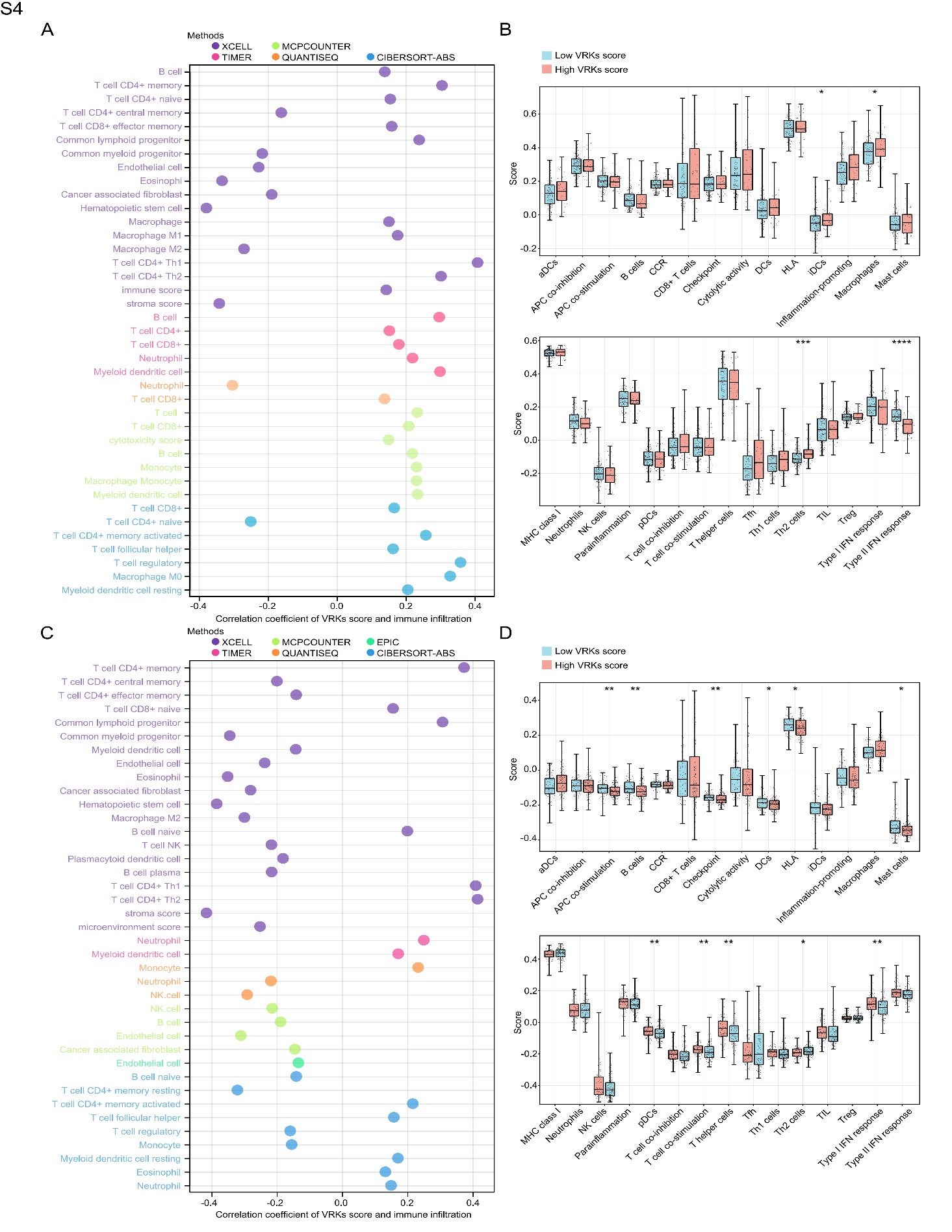


**Supplementary Figure 4.** The immune microenvironment analysis of the VRKs score. (A) The heatmap of immune cell infiltration and correlation with the VRKs score in the ICGC cohort. (B) The score based on single-sample Gene Set Enrichment Analysis (ssGSEA) of immune cell infiltration and function between high and low VRKs score groups in the ICGC cohort. (C) The heatmap of immune cell infiltration and correlation with the VRKs score in the GSE14520 cohort. (D) The score based on ssGSEA of immune cell infiltration and function between high and low VRKs score groups in the GSE14520 cohort. NK, natural killer; DC, dendritic cell; CCR, chemokine receptor; HLA, human leukocyte antigen; MHC, major histocompatibility complex; Tfh, follicular helper T cell; Th, helper T cell; Treg, regulatory T cell. **P*<0.05, ***P*<0.01, ****P*<0.001, *****P*<0.0001.


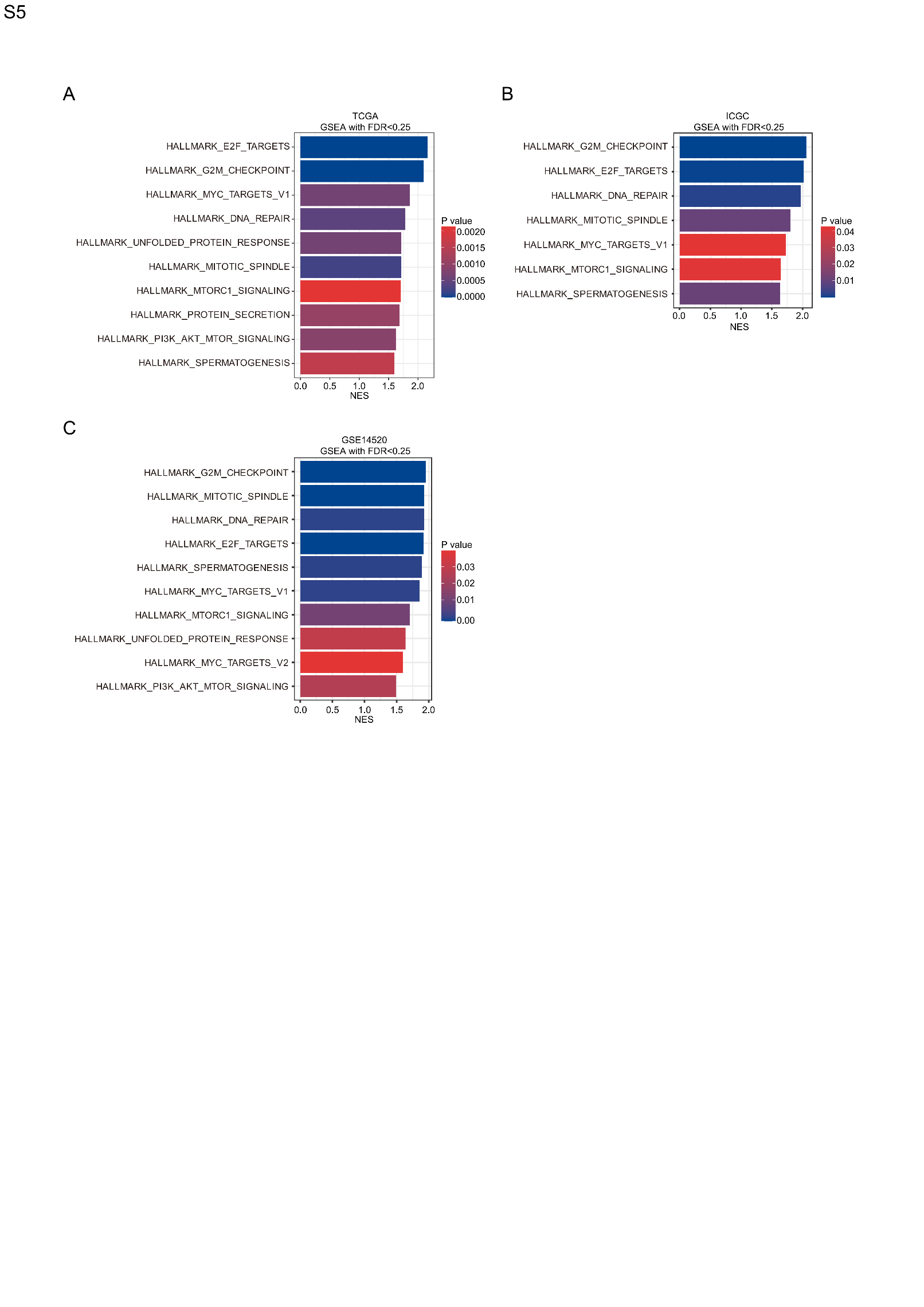


**Supplementary Figure 5.** The gene set enrichment analysis (GSEA) between high and low VRKs score groups. (A) The significant hallmark pathways with FDR<0.25 and *P*<0.5 based on GSEA between high and low VRKs score groups in the TCGA cohort. (B) The significant hallmark pathways with FDR<0.25 and *P*<0.5 based on GSEA between high and low VRKs score groups in the ICGC cohort. (C) The significant hallmark pathways with FDR<0.25 and *P*<0.5 based on GSEA between high and low VRKs score groups in the GSE14520 cohort. FDR, false discovery rate.


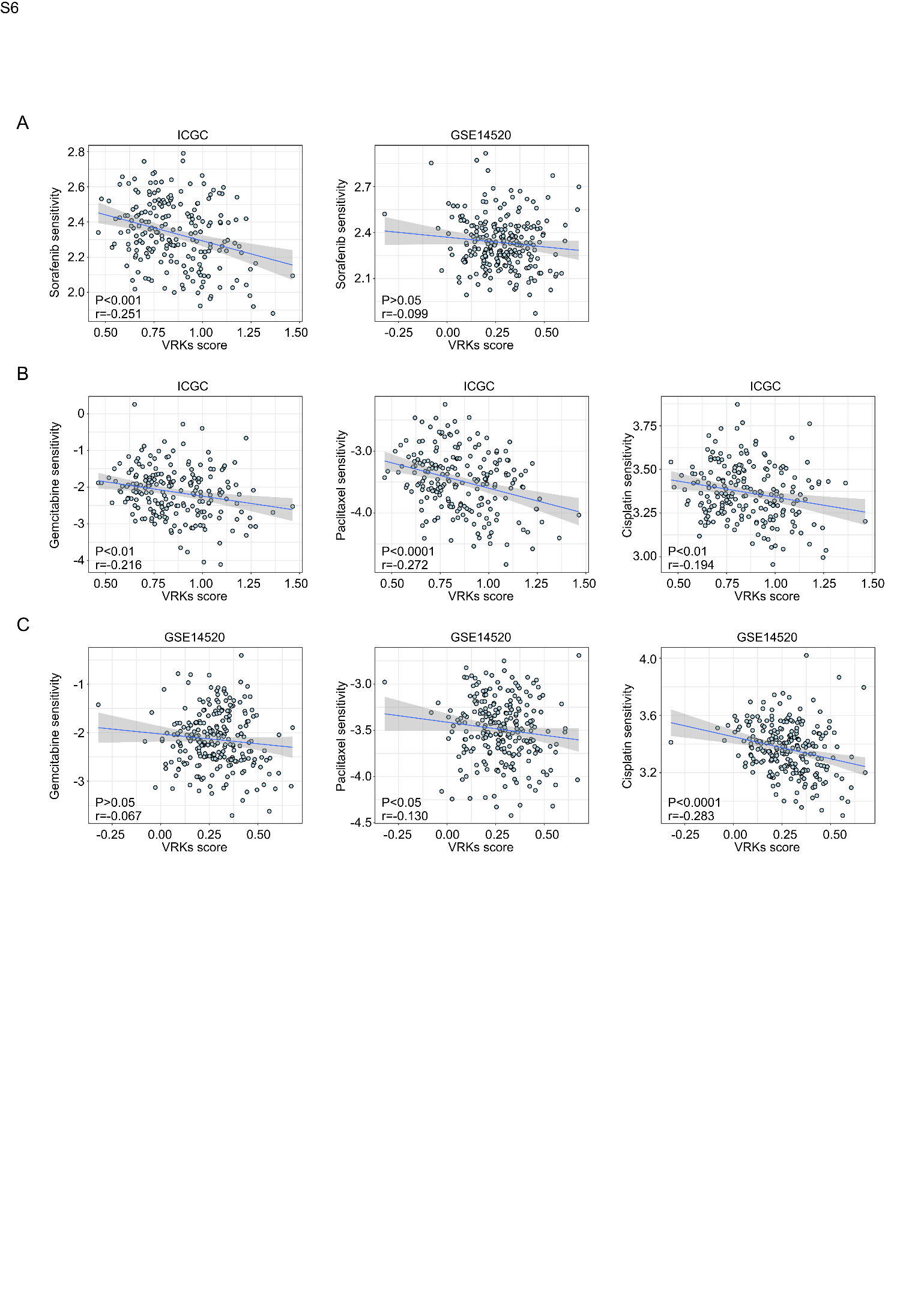


**Supplementary Figure 6.** The correlation analysis of the drug sensitivity and VRKs score. (A) The correlation analysis of the VRKs score and sorafenib sensitivity in ICGC and GSE14520 cohorts. (B) The correlation analysis of the VRKs score and gemcitabine, paclitaxel and cisplatin sensitivity in the ICGC cohort. (C) The correlation analysis of the VRKs score and gemcitabine, paclitaxel and cisplatin sensitivity in the GSE14520 cohort.


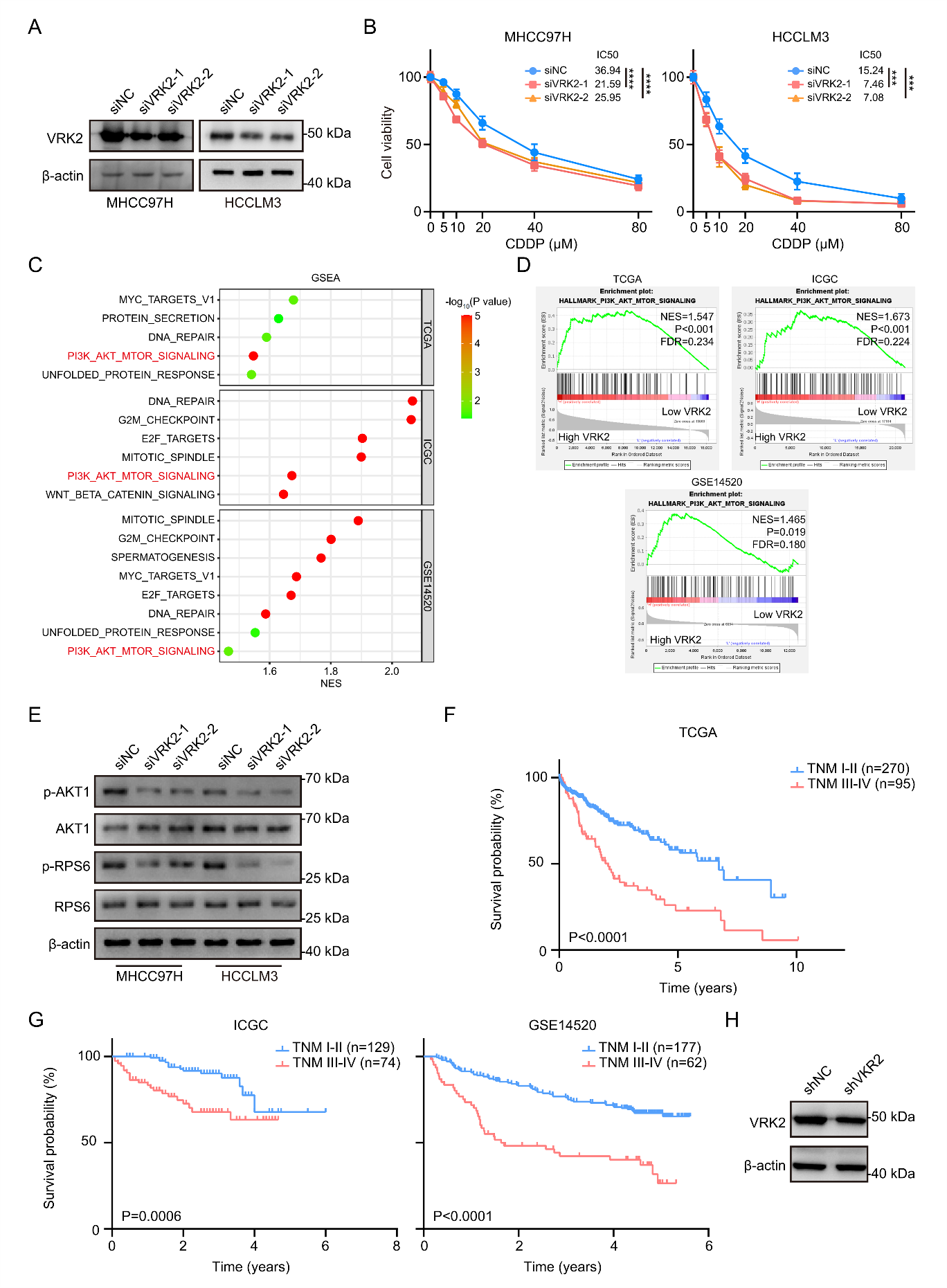


**Supplementary Figure 7.** VRK2 knockdown enhanced the sensitivity to cisplatin and decreased the AKT1 and RPS6 phosphorylation. (A) The western blotting validated VRK2 expression was successfully knocked down using siRNA in MHCC97H and HCCLM3cells. (B) VRK2 knockdown enhanced the sensitivity to cisplatin. (C) The GSEA analysis of TCGA, ICGC and GSE14520 cohorts. (D) The PI3K/AKT/mTOR results of GSEA analysis in TCGA, ICGC and GSE14520 cohorts. (E) Western blot results showed that VRK2 knockdown decreased the AKT1 and RPS6 phosphorylation. (F-G) The survival analysis between TNM I-II and III-IV groups in TCGA (F), ICGC and GSE14520 (G) cohorts. (H) Western blot results validated VRK2 expression was successfully knocked down using shRNA. GSEA, gene set enrichment analysis, cisplatin, CDDP; siRNA, small interfering RNA; shRNA, short hairpin RNA. **P*<0.05, ***P*<0.01, ****P*<0.001, *****P*<0.0001.

# Supplementary Tables

**Table S1 Designed sequences of siRNA used in the study**

| siRNA | Sequence |
| --- | --- |
| siVRK2-1 | Sense: AUAUAUCCAAGAUAAACCUG |
|  | Antisense: GGUUAUCUUGCAGAUAUGUG |
| siVRK2-2 | Sense: AAUAUUCCAAGAAUAACCUG |
|  | Antisense: CAAGUUUGAAUAUCUAUCUU |
| siNC | Sense: GGUUAUCUUGCAGAUGUAUGG |
|  | Antisense: ACGUGACGUUCGGAGAATT |
